# The Structure and Dynamics of *Bm*R1 Protein from *Brugia malayi*: *In* *Silico* Approaches

**DOI:** 10.3390/ijms150611082

**Published:** 2014-06-19

**Authors:** Bee Yin Khor, Gee Jun Tye, Theam Soon Lim, Rahmah Noordin, Yee Siew Choong

**Affiliations:** Institute for Research in Molecular Medicine, Universiti Sains Malaysia, Minden, Penang 11800, Malaysia; E-Mails: kbyin89@gmail.com (B.Y.K.); geejun@usm.my (G.J.T.); theamsoon@usm.my (T.S.L.); rahmah8485@gmail.com (R.N.)

**Keywords:** *Brugia malayi*, *Bm*R1 protein, protein structure prediction, epitope prediction, molecular dynamics simulation

## Abstract

*Brugia malayi* is a filarial nematode, which causes lymphatic filariasis in humans. In 1995, the disease has been identified by the World Health Organization (WHO) as one of the second leading causes of permanent and long-term disability and thus it is targeted for elimination by year 2020. Therefore, accurate filariasis diagnosis is important for management and elimination programs. A recombinant antigen (*Bm*R1) from the *Bm17DIII* gene product was used for antibody-based filariasis diagnosis in “Brugia Rapid”*.* However, the structure and dynamics of *Bm*R1 protein is yet to be elucidated. Here we study the three dimensional structure and dynamics of *Bm*R1 protein using comparative modeling, threading and *ab initio* protein structure prediction. The best predicted structure obtained via an *ab initio* method (Rosetta) was further refined and minimized. A total of 5 ns molecular dynamics simulation were performed to investigate the packing of the protein. Here we also identified three epitopes as potential antibody binding sites from the molecular dynamics average structure. The structure and epitopes obtained from this study can be used to design a binder specific against *Bm*R1, thus aiding future development of antigen-based filariasis diagnostics to complement the current diagnostics.

## 1. Introduction

Lymphatic filariasis (commonly known as elephantiasis) has infected 120 million people worldwide especially in developing and under-developed countries and approximately 1.3 billion people in 81 countries are at risk of infection. *Brugia malayi*, one of the causative agents of lymphatic filariasis, falls under the category of nematodes that infects human and animals. Infection occurs when the thread-liked parasitic filarial parasites are transmitted to humans through infected mosquitoes and develops into adult worms in human lymphatic vessels. In the year 2000, the WHO initiated a Global Program for Elimination of Lymphatic Filariasis (GPELF) with two main strategies: to stop the spread of infection (interrupting transmission) and to alleviate the suffering of the affected population (controlling morbidity) [1].

The availability of an easy on-site lymphatic filariasis diagnostic test which is rapid, affordable and accessible for disease management and therapy is one of the important factors for the elimination of lymphatic filariasis [2,3]. For brugian filariasis, one of the available diagnostics is the rapid immunochromatography detection of IgG4 antibody (Brugia Rapid). It is based on the recombinant antigen (*Bm*R1) expressed from *Bm17DIII* gene (GenBank: AF225296) [2,3,4]. Studies showed that the *Bm*R1 antigen is highly specific and sensitive for detection of IgG4 antibody in brugian filariasis [2,3,4]. Despite IgG4 being a good indicator of lymphatic filariasis, a sensitive and specific antigen-based detection test would also be an important alternative, which is currently not available. This antigen-based diagnostic would be a more direct test for active lymphatic filariasis infection. Thus, the sensitivity and specificity of *Bm*R1 antigen makes it a promising candidate for development of an antigen-based detection test.

In addition, identification of epitopes as possible antibody binding sites is important in leading to the development of such a test. The three-dimensional (3D) structure of the *Bm*R1 protein is essential in order to identify its epitopes, however, it has yet to be solved experimentally. Protein structure prediction is therefore the only way to predict the structure of *Bm*R1 from its amino acid sequence. Here, *in silico* studies were carried out to construct the predictive structure of *Bm*R1 and predict possible antibody epitopes of *Bm*R1. Our results showed that an *ab initio* method built the best model. A total of three epitopes have been identified from the average structure of molecular dynamics simulation.

## 2. Results and Discussion

### 2.1. Results

The molecular weight of the *Bm*R1 protein (206 amino acids) is 25 kDa. BLASTp results against non-redundant protein identified neither protein family nor conserved domains. Functional annotation by CD Search returned the same result as the BLASTp search. InterProScan, SMART and Pfam results have showed that the residue from 45–148 is a domain of unknown function 148 (DUF148). Pfam also indicated a match to baculovirus polyhedron protein, PEP C terminus from residues 116–160. Secondary structure prediction showed that *Bm*R1 sequence consists of 9–11 α-helices and 1–3 β sheets (Table 1).

**Table 1:** Secondary structure prediction by PSIPRED [5], Jpred3 [6], SSpro 4.0 [7] and PORTER [8]. Secondary structure calculation of average MD structure performed by STRIDE [9].

	1	11	21	31
**Sequence**	**M I K M N E K Y V K**	**E L I L L L L F A M**	**I Y T S L E S N C E**	**F W I E D D F H P F**
PSIPRED	- - - H H H H H H H	H H H H H H H H H H	H H H H H H H - - B	B B B - - - - - - -
Jpred3	- - - - - H H H H H	H H H H H H H H H H	H H H - - - - - - B	B B B - - - - - - -
SSpro	- - - - - H H H H H	H H H H H H H H H H	H H H H H H H - - -	B B B B - - - - - -
PORTER	- - - - - H H H H H	H H H H H H H H H H	H H H H H H H - - -	H H H - - - - - - -
STRIDE	- - - - H H H H H H	H H H H H H H H H H	H H H H H H H H H H	H H H H H - - - - -
	**41**	**51**	**61**	**71**
**Sequence**	**V P K S E E A R E E**	**Y C G F F K E M N L**	**S R N E L M D T I R**	**K W A S K Y G V L E**
PSIPRED	- - - - H H H H H H	H H H H H H H - - -	- H H H H H H H H H	H H H H H H H H H H
Jpred3	- - - - - - H H H H	H H H H H H H - - -	- H H H H H H H H H	H H H - - - - H H H
SSpro	- - - - H H H H H H	H H H H H H H H H -	- H H H H H H H H H	H H H H H - - H H H
PORTER	- - - - H H H H H H	H H H H H H H H - -	- H H H H H H H H H	H H H H H H H H H H
STRIDE	- - H H H H H H H H	H H H H H H H H - -	- - - - H H H H H H	H H H H H H - H H H
	**81**	**91**	**101**	**111**
**Sequence**	**Q F D N Y V D E E L**	**R Y E N M V Y D I F**	**K D K V N S T C G S**	**E K I K R T L F E I**
PSIPRED	H H H H H H H H H H	H H H H H H H H H H	H H H H - - - - - H	H H H H H H H H H H
Jpred3	H H H H H H - - - -	- - - - H H H H H H	H H - - - - - - - -	H H H H H H H H H H
SSpro	H H - - - - H H H H	H H H H H H H H H H	H - - - - - - - - H	H H H H H H H H H H
PORTER	H H H H H H H H H H	H H H H H H H H H H	H H H H - - - - - H	H H H H H H H H H H
STRIDE	H H H H H H - - - H	H H H H H H H H H H	H H H H H H H - - -	- H H H H H H H H H
	**121**	**131**	**141**	**151**
**Sequence**	**T D L L T D R D T A**	**Q Q T I Q T K I D E**	**I I N N L N E R E R**	**M E L T Q L W A I L**
PSIPRED	H H H H - - H H H H	H H H H H H H H H H	H H H - - - H H H H	H H H H H H H H H H
Jpred3	H H H H - - - H H H	H H H H H H H H H H	H H H H - - - - - H	H H H H H H H H H H
SSpro	H H H H - - H H H H	H H H H H H H H H H	H H H H H H H H H H	H H H H H H H H H H
PORTER	H H H H - - H H H H	H H H H H H H H H H	H H H H H H H H H H	H H H H H H H H H H
STRIDE	H H H - H H H H H H	H H H H H H H H H H	H H H H - - H H H H	H H H H H H H H H H
	**161**	**171**	**181**	**191**
**Sequence**	**G E E A I I E A Q D**	**K F E N G N S I W E**	**A V E N T T Q T D N**	**F K S E I V K D N D**
PSIPRED	H H H H H H H H H H	H H H - - - H H H H	H H H - - - - - - -	H H H H H H - - - -
Jpred3	- - H H H H H H H H	H H - - - - - H H H	H H H - - - - - - -	- H H H B - - - - -
SSpro	H H H H H H H H H H	H - - - - - - B B B	B B B - - - - - - -	- - H H H - - - - -
PORTER	H H H H H H H H H H	H H H - - - H H H H	H H H H - - - - - -	- H H H H H - - - -
STRIDE	- H H H H H H H H H	H H H H - - - H H H	H H H H - - - - - -	- - - H H H H H H H
	**201**			
**Sequence**	**K I L I S N**			
PSIPRED	B B B - - -			
Jpred3	B B B B - -			
SSpro	B B B B - -			
PORTER	- B B - - -			
STRIDE	- - - - - -			

H = α-helices; B = β-strand.

**Table 2:** Secondary structure calculation by STRIDE [9] on the models built by MODELLER 9v9 [10], QUARK [11], Robetta [12,13], Rosetta [14], I-TASSER [15] and Bhageerath [16].

	1	11	21	31
**Sequence**	**M I K M N E K Y V K**	**E L I L L L L F A M**	**I Y T S L E S N C E**	**F W I E D D F H P F**
MODELLER9v9	- - - - - - H H H -	H H H H H H H H H H	H H H H H H - - - -	- - - - - - - - - -
QUARK	- - - - - H H H H H	H H H H H H H H H H	H H H H H H H - - -	- - - - - - - - - -
Robetta	- - H H H H H H H H	H H H H H H H H H H	H H H H H H H - - -	- - - - - - - - - -
Rosetta	- - - - H H H H H H	H H H H H H H H H H	H H H H H H H H H H	H H H H H - - - - -
I-TASSER	- - - - - - H H H H	H H H H H H H H H -	- - H H H H H H H H	H H - - - - - - - -
Bhageerath	- - - - - H H H H H	H H H H H H H - - -	- - - - - - - - - -	- - - - - - - - - -
	**41**	**51**	**61**	**71**
**Sequence**	**V P K S E E A R E E**	**Y C G F F K E M N L**	**S R N E L M D T I R**	**K W A S K Y G V L E**
MODELLER9v9	- - - - H H H H H H	H H H H H H H H H H	H H H H H H H H H H	H H H H H H - H H H
QUARK	- - - - H H H H H H	H H H H H H H H - -	- H H H H H H H H H	H H H H H H - H H H
Robetta	- - - H H H H H H H	H H H H H H H H - -	- H H H H H H H H H	H H H H H H - H H H
Rosetta	- H H H H H H H H H	H H H H H H H H - -	- - - - H H H H H H	H H H H H - - H H H
I-TASSER	H H H H H H H H H H	H H H H H H H H - -	- H H H H H H H H H	H - - - H H H H H H
Bhageerath	- H H H H H H H H H	H H H H H H H H H H	H H H H - - - - - -	- - H H H H H H - H
	**81**	**91**	**101**	**111**
**Sequence**	**Q F D N Y V D E E L**	**R Y E N M V Y D I F**	**K D K V N S T C G S**	**E K I K R T L F E I**
MODELLER9v9	H H H H H H H H H H	H H H – H H H H H H	H H H H H H - - - H	H H - - H H H H H H
QUARK	H H H H H H H H H H	H H H H H H H H H H	H H H H H - - - - H	H H H H H H H H H H
Robetta	H H H H H H H H H H	H H H H H H H H H H	H H H H H H - - - H	H H H H H H H H H H
Rosetta	H H H H H H H H H H	H H H H H H H H H H	H H H H H H - - - H	H H H H H H H H H H
I-TASSER	H H H H H H H H - -	- H H H H H H H H H	H H H H H - - - - -	H H H H H H H H H H
Bhageerath	H H H H H H H H H H	H H H H H H H H H -	- - H H H H H H H H	H H H H H H H - - -
	**121**	**131**	**141**	**151**
**Sequence**	**T D L L T D R D T A**	**Q Q T I Q T K I D E**	**I I N N L N E R E R**	**M E L T Q L W A I L**
MODELLER9v9	H H H H H - H H H H	H H H H H H H H - H	H H H H H H H - H H	H H H H H H H H H H
QUARK	H H H H - - H H H H	H H H H H H H H H H	H H H H - - H H H H	H H H H H H H H H H
Robetta	H H H H H – H H H H	H H H H H H H H H H	H H H H - - H H H H	H H H H H H H H H H
Rosetta	H H H H H H H H H H	H H H H H H H H H H	H H H H - - H H H H	H H H H H H H H H H
I-TASSER	H H H H - - H H H H	H H H H H H H H H H	H H H - - - H H H H	H H H H H H H H H H
Bhageerath	- - - - - H H H H H	H H H H H H H H H H	- - - - - - - - - -	- - - - H H H H H H
	**161**	**171**	**181**	**191**
**Sequence**	**G E E A I I E A Q D**	**K F E N G N S I W E**	**A V E N T T Q T D N**	**F K S E I V K D N D**
MODELLER9v9	H H H H H H H H H H	H H H H H H H H - H	H H - - - - - - - -	- - H H H - - - - -
QUARK	H H H H H H H H H H	H H H H - - - H H H	H H H - - - - H H H	H H H H H H H - - -
Robetta	H H H H H H H H H H	H H H H - - - H H H	H H H - H H H H H H	- - - - H H H H - -
Rosetta	- H H H H H H H H H	H H H H - - - H H H	H H H H - - - - - -	- - - H H H H H H -
I-TASSER	- - - - - H H H H H	H H H - - - - - H H	H H H H H H H H - -	H H H H H H - - - -
Bhageerath	H H H H H H H H H H	H H H H - - - - - -	- - - - - - - - - -	- - - - - - - - - -
	**201**			
**Sequence**	**K I L I S N**			
MODELLER9v9	- - - - - -			
QUARK	- - - - - -			
Robetta	- H H H - -			
Rosetta	- - - - - -			
I-TASSER	- - - - - -			
Bhageerath	- - - - - -			

H = α-helices; B = β-strand.

For automated protein structure prediction, only CPHmodels 3.0, QUARK, Bhageerath, Robetta and I-TASSER were able to predict the structure of *Bm*R1. Secondary structure calculations by STRIDE [9] were done for the predicted structures to obtain the secondary structure information are showed in Table 2. The predicted structure from automated protein structure prediction, MODELLER 9v9 and Rosetta were further evaluated for backbone conformation and compatibility (Table 3). Overall, the evaluation data indicated that model built by Rosetta was the best structure with 97.5% of residues in the most favored region from the Ramachandran plot, 71.0% VERIFY 3D and 97% ERRAT scores.

**Table 3:** Model validation of structures predicted via comparative modeling, threading, *ab initio* method or combination of the approach.

Name	Approach	Ramachandran Plot [17]		VERIFY3D [18] (%)	ERRAT [19] (%)
		Residues in Most Favored Region (%)	Residues in Disallow Region		
MODELLER 9v9 [10]	Comparative modelling	87.3	-	16.4	58.6
CPHmodels 3.0 [20]	Comparative modelling	79.6	-	48.3	93.3
QUARK [11]	*Ab initio*	92.9	-	83.6	95.5
Robetta [12,13]	*Ab initio*	97.5	-	73.9	91.4
Rosetta [14]	*Ab initio*	98.5	-	71.0	97.0
I-TASSER [15]	Threading & *ab initio*	80.7	Ser 61	55.6	96.0
Bhageerath [16]	*Ab initio*	84.3	Met 20 Ser 24 Ile 142 Asp 102 Asn 176	33.3	61.4

**Figure 1:**
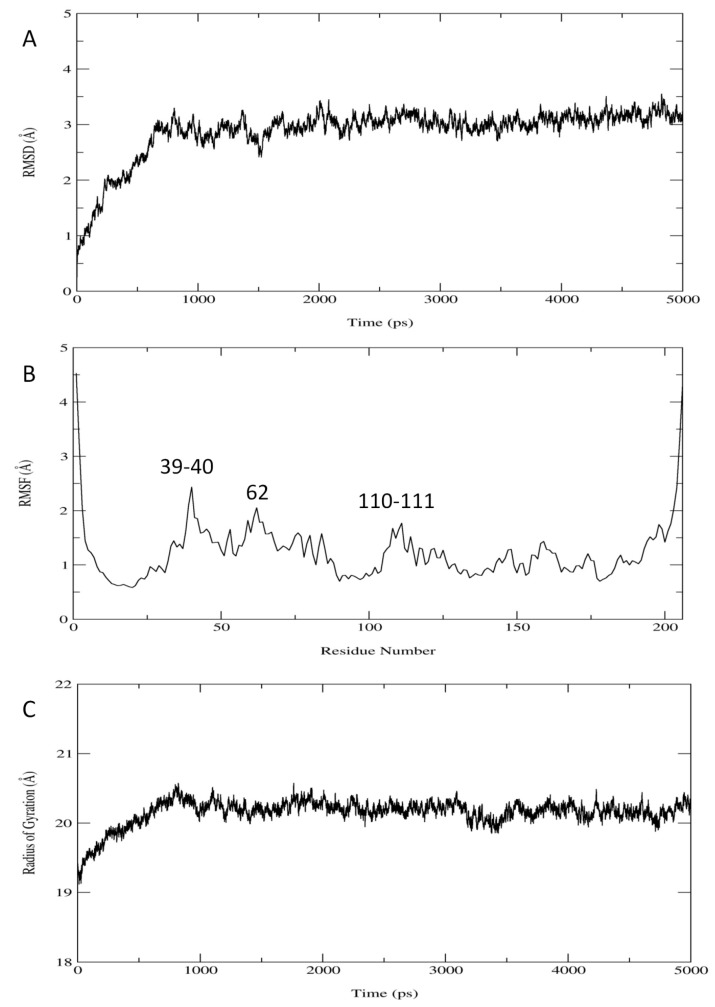
Analysis on (**A**) RMSD (**B**) RMSF and (**C**) Radius of gyration of *Bm*R1 protein during molecular dynamics simulation.

A 5 ns molecular dynamics (MD) simulation was performed with structure predicted by Rosetta. The root mean square deviation (RMSD) with respect to its starting structure shows that *Bm*R1 protein is stable after 1000 ps with an average RMSD of 2.9 ± 0.5 Å (Figure 1A). RMS fluctuation (RMSF) is approximately 4.5 Å at the *C*- and *N*-terminal and less than 2.5 Å for other residues (Figure 1B). Value for radius of gyration is also equilibrated after 1000 ps with the average of 20.2 ± 0.2 Å (Figure 1C). Average MD structure was created based on this analysis (1001 to 5000 ps). Secondary structure calculation on the average MD structure has showed similar patterns with the secondary structure prediction from PSIPRED [5], Jpred3 [6], SSpro 4.0 [7] and PORTER [8] in Table 1 and the initial structure (Rosetta) in Table 2, except the beta sheets regions (residue 30–33 and 201–204).

**Figure 2:**
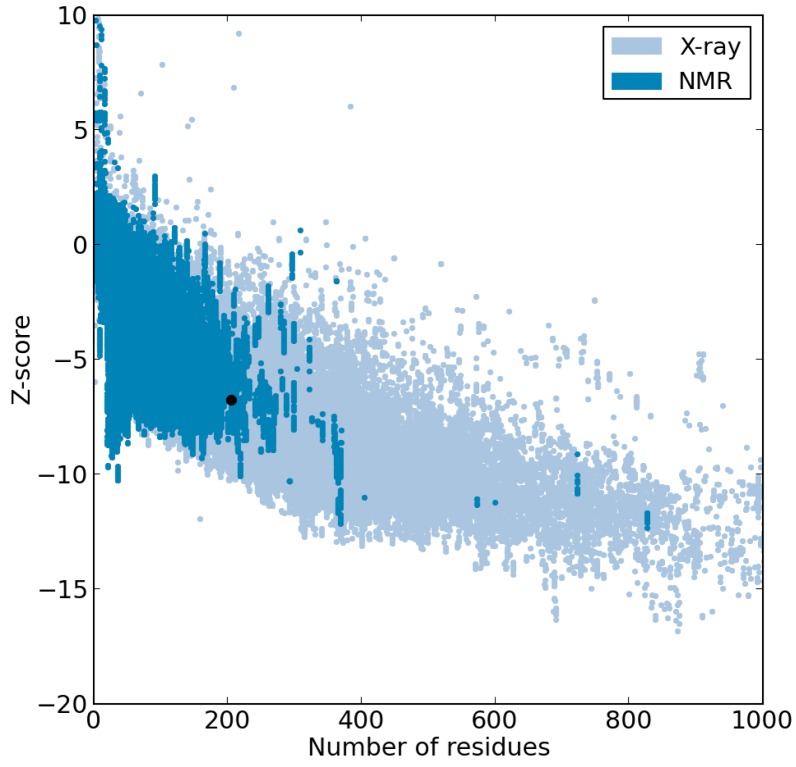
Prosa II Z-score plot of *Bm*R1 protein. The Z-score for modelled *Bm*R1 protein is represented as a black dot.

**Figure 3:**
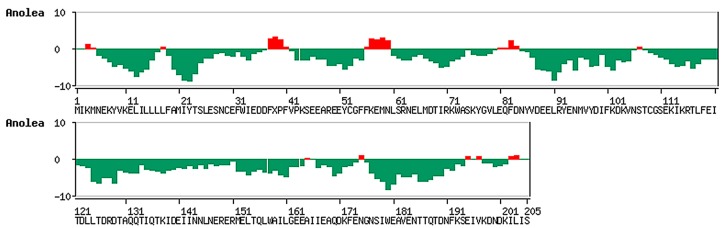
The packing quality of average MD structure analysed by ANOLEA [22]. High-energy amino acids show positive ANOLEA values (red bar) while low energy amino acids are with negative ANOLEA values (green bar).

The average MD structure was further analysed by Prosa II Z-score [21] to access the quality of the results. The Z-score value for average MD structure was −6.77, which is within the range observed for native set of proteins of same size (Figure 2). If the Z-score of a model structure is located outside the range of typically native proteins found by X-ray and NMR, it indicates an erroneous structure [21]. In addition, ANOLEA [22] was used to evaluate the packing quality of the modelled structure based on the non-local atomic interactions [23]. The ANOLEA program is able to assess the global quality of protein, observe local error and consequently gives the energy value for each amino acid of a protein [22,24]. Figure 3 shows the pseudo-energy profile for each amino acid. It appears that the high-energy zones (positive ANOLEA values; Figure 3) are located at the loops. From the result, most of the amino acids fall into the negative ANOLEA values (Figure 3), which indicates that the energy of amino acids is in a favorable state.

STRING [25] and PROSITE [26] were unable to analyse the function of *Bm*R1. ProFunc [27], which requires protein 3D structure as input, showed that *Bm*R1 has 23% sequence identity to PDB id 3KNT [28] and 3FHF [29]. PDBeMotif [30] and Motif Scan from ExPASy [31] showed that there are two *N*-glycosylation sites (residues 59–62 and 105–108), three protein kinase C phosphorylation sites (residues 68–70, 110–112 and 125–127) and six casein kinase II phosphorylation sites (residues 23–26, 27–30, 61–64, 116–119, 125–128 and 136–139) in the *Bm*R1 protein.

**Figure 4:**
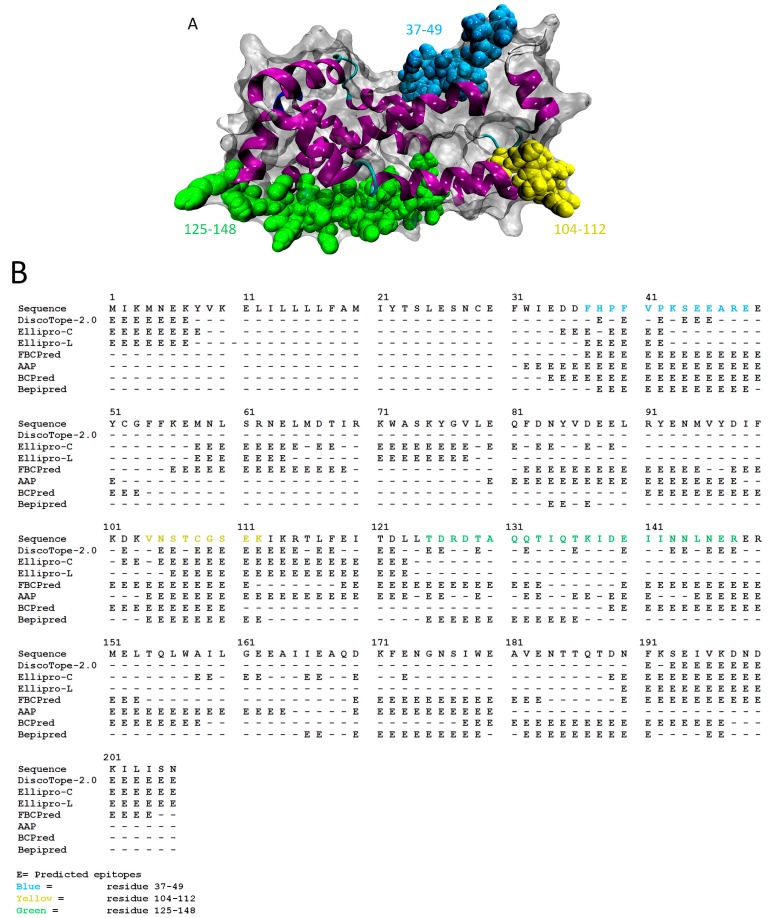
(**A**) Surface representation of built structure of average MD *Bm*R1 structure with predicted potential epitopes (residues 37–49, 104–112 and 125–148); (**B**) Predicted epitopes from Ellipro [32], DiscoTope-2.0 [36], FBCPred [33], AAP [34], BCPred [33] and Bepipred [35]. Ellipro-C represents predicted conformational epitopes and Ellipro-L represents predicted liner epitopes.

Surface representation of built average MD structure with predicted epitopes and binding sites is depicted in Figure 4A. Predicted linear epitopes (by Ellipro [32], FBCPred [33], AAP [34], BCPred [33] and Bepipred [35]) were overlapped with predicted conformational epitopes (by Ellipro [32] and DiscoTope-2.0 [36]). Based on these analyses, at least five servers predicted that residues 37–49, 104–112 and 193–197 (Figure 4B) are epitopes. Although epitope prediction for linear and conformational epitopes showed comparable results, the sequence 193–197 was not selected as potential epitopes as there are only 5 residues [37]. Protein binding site prediction of *Bm*R1 protein by ProBis [38] showed structurally conserved sequences, located at 45–48, 70–73 and 126–143. Based on the result from COACH server, residues 18, 66, 72, 76, 126, 128, 130, 133, 135, 138–140, 143, 147 and 149 are predicted as potential protein-ligand binding sites. Therefore, regions 125–148 was taken into consideration as a potential binding site. As a conclusion, we report three potential epitopes (sequences 37–49, 104–112 and 125–148).

### 2.2. Discussion

*Bm17DIII* gene product of *B. malayi* in this study is the *Bm*R1 protein with 206 amino acids. The structure prediction for *Bm*R1 is challenging for several reasons. First, no putative conserved domains and functional annotation were identified. Conserved domains are important for elucidating the protein’s function; Secondly, the sequence identity of the *Bm*R1 protein with available structures in PDB is less than 30%. This increases the probability of errors in predicted models, such as errors in side-chain packing, distortions and shifts in correctly aligned regions, errors in regions without a template, errors due to misalignment and incorrect templates [39].

Protein structures can be modeled via comparative method (both comparative modeling and threading) that depends on known protein structures or by *ab initio*, that relies on amino acid sequences. To date, comparative modeling is the most successful and accurate method as evolutionarily related proteins usually share a similar structure (sequence identity > 30%) [40,41]. However, searching for homologous proteins is difficult when the sequence identity is low or known as the “twilight-zone”, where the sequence identity falls between 10%–30% [42]. When confronted with this problem, threading or *ab initio* is an alternate method to obtain the protein structure [43]. In this study, comparative and *ab initio* methods were performed in order to obtain the most accurate structure. For comparative modeling, a total of 250 structures were generated by MODELLER 9v9 [10]. After subsequent steps of secondary structure restraints and loop refinement, the best model was selected based on high DOPE score and low MODELLER objective function (molpdf) as lower values of the molpdf indicate more accurate models. The molpdf measures how well the model satisfies the input spatial restraints [44]. The optimized model from comparative modeling (MODELLER 9v9 [10]) was evaluated (PROCHECK Ramachandran plot [17], VERIFY3D [18] and ERRAT [19]). A Ramachandran plot showed that even though the residues in the most favorable region are 87.3%, the VERIFY3D (16.43%) and ERRAT score (58.6%) were low (Table 3). VERIFY3D score of a satisfactory predicted model is expected to have score more than 80% and a value of ~95% ERRAT score indicates high resolution (approximately 2.5–3.0 Å) [19,45]. Due to the unsatisfactory evaluation result, *ab initio* approach by Rosetta and nine web servers were employed to obtain the structure for *Bm*R1 protein. For automated protein structure prediction, SWISS-MODEL [46], 3D-JIGSAW [47], ESyPred3D [48], Geno3D [49] failed as there is no suitable templates which are similar to known structure. Out of nine servers, only CPHmodels 3.0 [20], QUARK [11], Robetta [12,13], I-TASSER [15] and Bhageerath [16] were able to predict the structure of *Bm*R1. Evaluation results for the structures obtained were compared (Table 3).

The percentage of residues in the allowed regions was expected to be more than 90% for a good model. Results from a Ramachandran plot showed more than 90% of the residues built from Robetta, and Rosetta and QUARK were in the most favorable region. Structure obtained by Rosetta showed that 98.5% of the residues were in allowed regions and none were in the disallowed region in the Ramachandran plot. Even though the VERIFY3D score of Rosetta (71.01%) is slightly lower than the model by Robetta (73.91%) and QUARK (83.57%), the overall quality factor (ERRAT) value is the highest (97%) and is within the accepted range. Therefore, the structure built by Rosetta was the best amongst others (Table 3). The G-factor score, which indicates the overall normality of a model, obtained from Rosetta is 0.48. This shows that the molecular geometry of the structure is stereochemically reasonable. The overall quality factor (ERRAT) value is 97% thus concluding that the built model would have a resolution of not more than 3 Å. A VERIFY3D value of 71.0% showed that the quality of predicted structure was expected to be satisfactory. Thus, the overall model validation showed that the structure predicted by Rosetta was reasonable.

When the sequence identity between two proteins is less than 30%, it is difficult to discriminate between related or non-related protein. In this case, the secondary structure of the protein could provide valuable information for the detection of related protein 3D structure. Secondary protein structure is important in analysing tertiary protein structure predictions as it represents the local conformation of amino acids into regular structures. Proteins with low sequence similarity are also likely to have higher similarity in their secondary structure information if they are from the same structural class [50]. Thus, secondary protein structure is utilized as a feature for tertiary protein structure prediction [51,52]. Secondary protein structure predictions based on protein sequence by PSIPRED [5], Jpred3 [6], SSpro 4.0 [7] and PORTER [8] showed in Table 1 were compared to the secondary protein structure calculation using STRIDE [9]. Secondary structure calculation of all the predicted structures (QUARK [11], Robetta [12,13], Rosetta [14], Bhageerath [16], I-TASSER [15]) was compared in Table 2. CPHmodels 3.0 [20] was not included as the protein generated was less than 206 amino acids. All predicted protein structures have similarity in their secondary structure except for structure predicted using Bhageerath. In this study, results from secondary structure prediction of the *Bm*R1 sequence and the STRIDE calculation of the average MD structure showed that the protein consists of 9 helices. Beta sheet was not detected for both the starting structure and average MD structure may be due to the lower reliability value of prediction accuracy for the beta sheet. The reliability value obtained for the beta sheet is within the scores of 5 or lower (the value ranges from 0–10 with higher value indicating better reliability).

Based on the secondary protein structure, the 25 kDa *Bm*R1 protein is a helix-rich protein. Fatty acid and retinol (Vitamin A)-binding (FAR) protein, which appear to be confined to nematodes, are relatively small in size (~20 kDa) and rich in alpha-helices [53]. The similarities showed a potential relationship between *Bm*R1 protein and FAR protein. In addition, multiple sequence alignment (MSA) of *Bm*R1 protein with 4 different parasitic FAR proteins of nematodes by T-Coffee [54] showed approximately 28% similarity. *Brugia malayi* (Bm-FAR-1), *Brugia pahangi* (Bp-FAR-1), *Wuchereria bancrofti* (Wb-FAR-1) and *Loa loa* (Ll-FAR-1) were chosen for MSA as a distance-based analysis of FAR protein. Garofalo *et al.*, [53] showed that the FAR proteins were from two main clusters but only FAR proteins from the same cluster as *B. malayi* was further studied here. Studies suggested that the FAR protein may play a crucial role in the life cycle, development and reproduction of nematodes and infection [55,56]. The FAR protein scavenges fatty acids and retinols from the host for the survival of the parasite [55,56]. Nematodes require fatty acids and retinol for lipid biosynthesis and assembly of macromolecular structures. However, they are unable to synthesize those metabolites by themselves thus making this a logical possibility for survival [57]. FAR protein not only helps nematodes in obtaining lipid from its host but also to infect the host and inhibit host defense mechanism [55]. The suggested relationship of *Bm*R1 protein and FAR protein remains relevant as there is no data showing the function of *Bm*R1 protein yet. In 2008, Moreno and Geary analysed the excretory-secretory products (ESP) of adult female, adult male and microfilariae (Mf) of *B. malayi* [58]. A total of 76 proteins in Mf, including recombinant antigen R1 (similar in sequence to *Bm*R1) were analysed. From the analysis with Blast2GO [59] recombinant antigen R1 remained as a protein with no annotated function but was the most abundantly expressed in Mf. A year later, Bennuru and colleagues also failed to resolve the function of recombinant antigen R1 [60]. The function of *Bm*R1 remain unknown in our analysis with Blast2GO (data not shown).

Protein function analysis from ProFunc [27] showed 23% of similarity to 3KNT and 3FHF, which are classified as *N*-glycosylase. There are two *N*-glycosylation sites detected in *Bm*R1 protein at residue 59–62 and 105–108. Some FAR proteins have a casein kinase II phosphorylation site [56,61]. Analysis results from PDBeMotif [30] and Motif Scan [31] showed that *Bm*R1 protein contains a site (residues 61–64) that is conserved in known FAR proteins [62].

The structure from Rosetta was used as a starting structure for a 5 ns MD simulation in the presence of water. In order to evaluate the stability of the built *Bm*R1 protein, the dynamics of the protein was studied. RMSF analysis from MD trajectories showed that the most flexible residues are located at both *C*- and *N*-terminal of the protein. The radius of gyration and RMSD of the protein area plateaued after 1000 ps, showing no significant changes to the overall structure. The average structure with a MD simulation time of 1001 to 5000 ps was evaluated, showing good quality secondary structure and overall packing (Figure 1).

Regions predicted by at least five out of seven servers were selected as epitopes. Epitope prediction was carried out to identify the binding site of an antigen, which is usually located on the surface, loops and turns of an antigen. In 1986, Novotny and colleagues proposed that surface exposure of protein was the reason for the contact with antigen- combining sites [63]. In the same year, Barlow and colleagues found a good correlation between epitopes and protein regions protruding from protein’s globular surface [64]. Residues must be located on the surface of antigens in order to be recognized and accessible for interaction by antibodies [65]. Thus, surface exposure and accessibility was taken into consideration for epitope prediction (e.g., CEP [65], DiscoTope-2.0 [36], LEPS [66]). Surface accessibility was considered when predicting potential epitopes in both Ellipro [32] and DiscoTope-2.0 [36]. For Ellipro [32], a protrusion index (PI, Table S1) was given to each residue. PI was defined as percentage of protein atoms enclosed in the ellipsoid. Regions with high protrusion index values were identified as potential conformational epitopes [32]. In DiscoTope-2.0 [36], overall prediction scores which included surface measures were analysed. Linear and conformational epitope predictions were also carried out here. However, the final selection of potential epitopes depends on the number of residues to form an epitope. The sequence 193–197 was not taken into consideration as it contains only 5 residues. A study showed that the total number of amino acid residues per epitope ranged from 9 to 22 residues for antibodies [37]. The predicted epitope of sequence 37–49 also included residues 45–48 which were predicted as the structurally conserved residues and location of putative binding sites by ProBis [38] making it a sound predicted epitope. ProBis and COACH predicted sequence 125–148 as a potential epitope. From these data we suggest 3 potential epitopes sequences 37–49, 104–112 and 125–148. These epitopes may thus lead to the generation of designer antibodies specific to *Bm*R1 protein. 

## 3. Methods

### 3.1. Sequence Analysis

The amino acid sequence of *Bm*R1 antigen was retrieved from GenBank (accession number AF225296). ProtParam from Expert Protein Analysis System (ExPASy) Proteomics Server [31] was implemented to calculate the protein molecular weight. The protein has a total of 206 amino acids and was subjected to Basic Local Alignment Search Tool (BLAST) on the NCBI server against non-redundant protein sequences to determine protein family. Templates identification for comparative modeling was performed using protein-protein BLAST (BLASTp) against RCSB Protein Data Bank (PDB) with default parameters. Secondary structure prediction on *Bm*R1 was performed by PSIPRED [5], Jpred3 [6], SSpro 4.0 [7] and PORTER [8]. Function annotation and identification of the conserved domain of *Bm*R1 were carried out using databases such as Conserved Domain Search Service (CD Search) [67,68], InterProScan [69], SMART [70] and Proteins Families database (Pfam) [71]. For protein functional analysis, ProFunc [27], STRING [25], PROSITE [26], PDBeMotif [30] and Motif Scan from ExPASy [31] were used. Linear epitope prediction on *Bm*R1 protein sequence was carried out by FBCPred [33], AAP [34], BCPred [33] and Bepipred [35]. T-Coffee (Tree-based consistency objective function for alignment evaluation) [54] was used for multiple sequence alignment.

### 3.2. Structural Prediction and Evaluation

The structure of *Bm*R1 was modelled via comparative modeling, threading and *ab initio* approaches. In comparative modeling by MODELLER 9v9 [10], a total of 250 initial models were generated from multiple templates (PDB id: 2G3Y, 2IE8 [72], 3QOE [73], 1V32, 2E87, 2R3V [74] and 3I4Q), followed by secondary structure restraints and loop refinement using MODELLER 9v9 [10]. DOPE score and molpdf were used to evaluate the models. Best structure with high DOPE score and low molPDF was chosen. Protein prediction by *ab initio* approach was carried out by Rosetta [14]. Fragment libraries of three- and nine-residue were generated by Robetta fragment server [12] and those models were assembled by fragment insertion [75]. Five hundred initial structures were created using AbinitioRelax command with the three- and nine-residue fragments as input. AbinitioRelax is the combination of *ab initio* folding and refinement by Rosetta full-atom force field (Relax) [14]. Automated server (SWISS-MODEL [46], 3D-JIGSAW [47], ESyPred3D [48], Geno3D [49], CPHmodels 3.0 [20] QUARK [11], Bhageerath [16], Robetta [12,13] and I-TASSER [15]) with default parameters were also employed to predict the structure of *Bm*R1.

All predicted structures were sent for secondary structure calculation using STRIDE [9] and were evaluated with PROCHECK Ramachandran plot [17], VERIFY3D [18] and ERRAT [19]. Ramachandran plot was obtained for backbone conformation evaluation [17]. VERIFY3D was used to determine the compatibility of an atomic model (3D) with its own amino acid sequence (1D) [18]. A higher score indicates high quality of a structure. ERRAT is to analyse the statistics of non-bonded interactions between different atom types.

### 3.3. Minimization and Molecular Dynamics Simulation

The protein model with the best validation value obtained from protein structure prediction subsequently underwent energy minimization by the Sander module from AMBER11 [76] with AMBER ff03 force field. The protein was solvated with TIP3P water in a truncated octahedron periodic box with 10 Å distance from the edge of the box. The solvated system was neutralized by 19 sodium ions (Na^+^). The system has a total of 37,223 atoms. The protein was restrained during the first stage of minimization with 300 kcal/mol restraint force. For stage 2 of the minimization, the entire system was minimized. The temperature of the system was gradually heated to 300 K over 20 ps. The system was subsequently equilibrated at 300 K over 60 ps during the NVT equilibration. Temperature was controlled by Langevin thermostat [77]. Finally, a total of 5000 ps MD simulation at 300 K and 1 atm was carried out. SHAKE algorithm [78] was turned on throughout the MD simulation to constrain bonds involving hydrogen.

Secondary structure calculation of the average MD structure was performed by STRIDE [9]. The structure was validated through PROCHECK Ramachandran Plot [17], VERIFY3D [18], ERRAT [19], Prosa II Z-score [21] and ANOLEA [22]. Conformational epitope prediction by Ellipro [32] and DiscoTope-2.0 [36] was performed on the average structure from MD simulation. The protein binding site of the *Bm*R1 protein was predicted by ProBis [38] and COACH [79].

## 4. Conclusions

In this study, *Bm*R1 structure predicted via *ab initio* method (Rosetta) produced a quality and reliable structure. Furthermore, the average structure obtained from molecular dynamics simulation also showed overall good secondary and 3D packing. A total of three potential epitopes were identified leading to the possibility of future designer antigen-based detection test and -specific binders capable for therapy using *Bm*R1 as the targeted antigen.

## Supplementary Files

Supplementary File 1 Supplementary Information (PDF, 561 KB)
