# Supplementary material for: The Structure and Dynamics of BmR1 Protein from Brugia malayi: In Silico Approaches
Source: Int J Mol Sci. 2014 Jun 19;15(6):11082–99. doi: 10.3390/ijms150611082 (PMC4100200; doi:10.3390/ijms150611082)
Supplement: Supplementary File 1 — Supplementary Information (PDF, 561 KB) [file ijms-15-11082-s001.pdf]

Supplementary Information

Table S1. Protrusion Index (PI) from Elipro [32] for the predicted epitopes (Blue = residue 37–49, Yellow = 104–112 and Green = residue 125–148).

Residue Number	Residue Name	Protrusion Index (PI)
37	PHE	0.340
38	HIS	0.859
39	PRO	0.908
40	PHE	0.976
41	VAL	0.786
42	PRO	0.728
43	LYS	0.369
44	SER	0.398
45	GLU	0.646
46	GLU	0.398
47	ALA	0.175
48	ARG	0.311
49	GLU	0.451
104	VAL	0.451
105	ASN	0.597
106	SER	0.786
107	THR	0.762
108	CYS	0.728
109	GLY	0.908
110	SER	0.956
111	GLU	0.981
112	LYS	0.937
125	THR	0.597
126	ASP	0.549
127	ARG	0.126
128	ASP	0.451
129	THR	0.597
130	ALA	0.311
131	GLN	0.175
132	GLN	0.549
133	THR	0.510
134	LYS	0.204
135	ILE	0.291
136	ASP	0.597
137	LYS	0.549
138	ILE	0.272
139	ASP	0.549
140	GLU	0.786
141	ILE	0.510
142	ILE	0.398
143	ASN	0.786
144	ASN	0.589
145	LEU	0.646

Table S1. *Cont.*

Residue Number	Residue Name	Protrusion Index (PI)
146	ASN	0.835
147	GLU	0.859
148	ARG	0.917

© 2014 by the authors; licensee MDPI, Basel, Switzerland. This article is an open access article distributed under the terms and conditions of the Creative Commons Attribution license (http://creativecommons.org/licenses/by/3.0/).
